# The mechanosensitive channel TMEM63B contributes to noxious heat sensation in the dorsal root ganglia

**DOI:** 10.1371/journal.pbio.3004060

**Published:** 2026-10-16

**Authors:** Chang Ye, Yan-Yu Zang, Gui-Fang Duan, Jiang Chen, Dan Wu, Dan Wang, Changlin Li, Yun Xu, Tianjiao Xia, Xiaoping Gu, Yun Stone Shi

**Affiliations:** 1 Department of Anesthesiology, Nanjing Drum Tower Hospital, Affiliated Hospital of Medical School, Nanjing University, Nanjing, China; 2 Guangdong Institute of Intelligence Science and Technology, Zhuhai, China; 3 Ministry of Education Key Laboratory of Model Animal for Disease Study, Model Animal Research Center, Medical School, Nanjing University, Nanjing, China; 4 State Key Laboratory of Natural and Biomimetic Drugs, School of Pharmaceutical Sciences, Peking University Health Science Center, Beijing, China; 5 Department of Neurology, Nanjing Drum Tower Hospital, Affiliated Hospital of Medical School, Nanjing University, Nanjing, China; Columbia University Irving Medical Center, UNITED STATES OF AMERICA

## Abstract

In mammals, heat sensation is primarily mediated by thermosensitive channels, particularly transient receptor potential (TRP) channels, in dorsal root ganglion (DRG) neurons. However, whether additional channels contribute to noxious heat detection remains unclear. Here, we investigated the role of the mechanosensitive cation channel TMEM63B in DRG neurons and unexpectedly discovered its involvement in noxious thermosensation rather than mechanosensation. TMEM63B was broadly expressed in DRG neurons, and its global knockout or DRG-specific conditional knockout markedly reduced heat sensitivity. Deletion of TMEM63B significantly attenuated heat-induced calcium responses and currents in DRG neurons and elevated the temperature threshold for action potential initiation. Recombinant TMEM63B exhibited moderate heat response in calcium imaging assays. Notably, in the presence of arachidonic acid, TMEM63B was strongly activated by heat, suggesting that its thermosensitivity is modulated by the membrane lipid environment. Together, these findings identify TMEM63B as a novel channel contributing to heat sensation and reveal a lipid-dependent modulation of its channel activity.

## Abstract 2

Heat sensation is primarily mediated by thermosensitive channels in mammals, however whether additional channels contribute to noxious heat detection remains unclear. This study shows that the mechanosensitive cation channel TMEM63B contributes to heat sensation through a unique mechanism modulated by the membrane lipid environment.

## Introduction

Nociception plays a critical role in protecting the body from injury. Noxious stimuli are detected by primary sensory neurons in dorsal root ganglia (DRG), which transmit signals via secondary neurons in the spinal dorsal horn to the central nervous system (CNS) [1,2]. These stimuli are sensed by specialized ion channels or receptors, expressed in DRG neurons. To date, numerous molecular components for nociceptive sensation have been identified. The classic heat sensor, TRPV1, responds to heat and chemicals such as protons and capsaicin [3–6]. Other thermosensitive TRP channels, including TRPV4, TRPM2 and TRPM3, also detect warm and heat stimuli [7–11], while TRPM8 and TRPA1 mediate cold sensation [12–15]. Mechanical pain and gentle touch are primarily transduced by Piezo2 [16–18]. Additionally, polymodally-gated TRPA1, acid-sensing ion channels (ASICs), and ATP-gated P2X receptors are involved in peripheral sensitization and chronic pain pathogenesis [19–22]. Beyond ion channels, other membrane receptors, such as G-protein (GPCRs) and receptor tyrosine kinases (RTKs) related signaling, also play indispensable roles in noxious stimulus sensation [23–25].

Notably, multiple receptors often contribute to the detection of a single noxious stimulus. For instance, TRPV1, TRPM3, and TRPA1 have all been implicated in noxious heat sensation [26]. However, in temperature choice experiments, mice lacking all three channels still avoid noxious heat [26], suggesting the existence of additional molecular component for heat transduction. Indeed, when TRPV1, TRPM3, and TRPA1 are pharmacologically blocked, TRPM2 deletion further reduces the number of heat-responsive DRG neurons [27]. The calcium-activated chloride channel ANO1 has also been reported to modulate noxious heat sensitivity, either as a direct sensor [28] or through regulating TRPV1 activity [29]. Beyond these ion channels, TMC6 has recently been shown to function as a Gαq-coupled GPCR-like receptor that specifically mediates noxious heat sensation [23]. This seemingly redundant mechanism may ensure that animals remain alert to noxious heat across diverse environmental contexts [26].

TMEM63B is a recently discovered nonselective cation channel permeable to monovalent cations Na^+^, K^+^ and divalent cations Mg^2+^ and Ca^2+^ [30,31]. It is osmosensitive [30–32] and activated by mechanical stimuli [33]. Structural and functional studies further reveal its role as a lipid scramblase, regulating phospholipid redistribution on cell membrane or organelle membrane [34,35]. While the molecular mechanisms underlying TMEM63s activation are still being elucidated through structural and functional analyses [36–38], studies using genetically modified animals have begun to uncover its diverse physiological roles. In mice, TMEM63B is essential for auditory function; its knockout leads to deafness due to the loss of cochlear outer hair cells [30]. In alveolar type II epithelial cells, TMEM63B senses inspiration-induced mechanical stretch and regulates pulmonary surfactant secretion [39,40]. TMEM63B also participates in endocrine signaling, modulating thyroid hormone secretion in follicular cells [41] and insulin release in pancreatic β-cells [42]. TMEM63B in intestinal stem cells emerges as a critical regulator of gastrointestinal motility and digestive function [43]. Within the CNS, TMEM63B acts as an osmosensor in interoceptive neurons, where it is crucial for thirst sensation [44,45]. Additionally, in olfactory interneurons, TMEM63B channels respond to plasma membrane tension changes, driving Ca^2+^-dependent neuronal migration [46].

In our previous study, we demonstrated that TMEM63B protects outer hair cells in the inner ear from degeneration, likely through its osmosensitive properties [30]. Interestingly, TMEM63B is also highly expressed in spiral ganglion neurons in the cochlea [30]. This observation prompted us to investigate whether it might be broadly expressed in sensory ganglia such as the DRG and contribute to somatosensation.

## Results

### TMEM63B is widely expressed in primary sensory neurons

To investigate the role of TMEM63B in somatosensation, we analyzed its expression pattern in DRG neurons using *Tmem63b*^*HA-fl*^ mice, which allow for both HA-tagged TMEM63B (HA-TMEM63B) detection and conditional knockout (cKO) [30]. HA-immunostaining revealed TMEM63B expression in 54% of DRG neurons (917 of 1,711 neurons; Fig 1A), with significant localization in small, medium, and large DRG neurons (Fig 1B). To precisely define the sensory neuronal subpopulations expressing TMEM63B, we performed a quantitative co-localization analysis of HA-TMEM63B with well-established DRG markers (Fig 1C and 1D). Consistent with the size distribution (Fig 1B), TMEM63B was abundantly expressed in approximately half of both CGRP-positive peptidergic neurons (50.24 ± 6.09%) and IB4-binding nonpeptidergic neurons (50.72 ± 6.89%), which represent two major classes of unmyelinated C-fiber nociceptors. TMEM63B was also widely expressed in NFH-positive neurons (65.96 ± 3.68%), which correspond to large-diameter myelinated sensory neurons. Notably, a substantial proportion of TRPV1-positive noxious heat sensors expressed TMEM63B (59.38 ± 5.29%).

**Fig 1:**
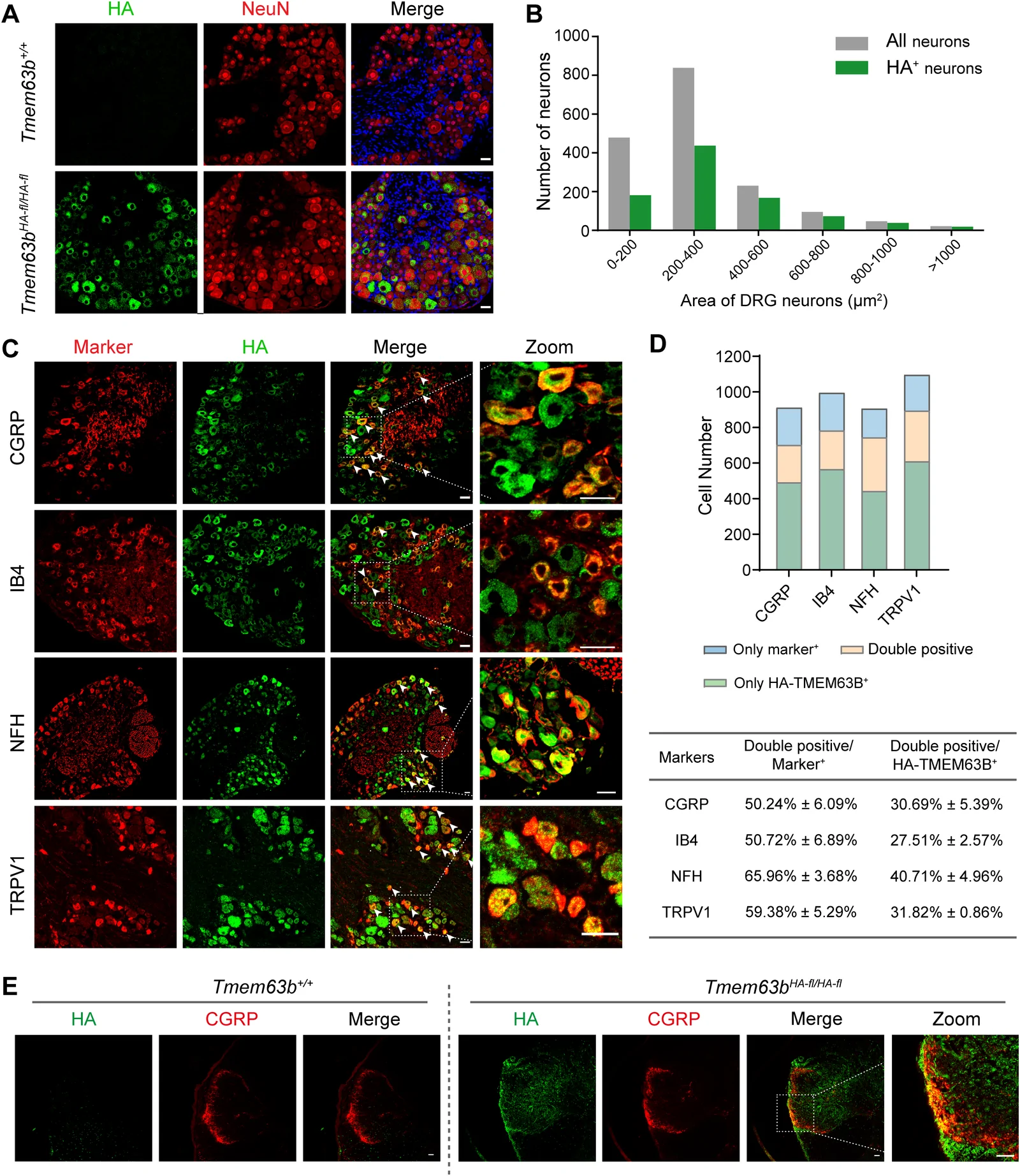
HA-TMEM63B is expressed in DRG neurons. **(A)** Immunofluorescent images of a DRG slice. Staining of HA (green) indicated expression of TMEM63B in primary sensory neurons labeled by NeuN (red). Scale bars: 30 μm. **(B)** Quantification of immunostaining data indicated the distribution of HA-TMEM63B positive cells in DRG neurons with different sizes. **(C, D)** Representative co-immunostaining images (C) and quantification of co-localization (D) for HA (green) and specific DRG markers (CGRP, IB4, NFH, and TRPV1; red). Scale bar: 30 μm. **(E)** Co-localization of HA-TMEM63B (green) and CGRP (red) in spinal dorsal horn. Scale bars: 30 μm. The data underlying this Figure can be found in S1 Data.

Conversely, within the TMEM63B-expressing population, 30.69 ± 5.39% co-localized with CGRP, 27.51 ± 2.57% with IB4, 40.71 ± 4.96% with NFH, and 31.82 ± 0.86% with TRPV1. Beyond the DRG soma, we also observed that HA-TMEM63B co-localized with CGRP in the central terminals of primary afferents within the spinal dorsal horn (Fig 1E).

Taken together, these results demonstrate that TMEM63B is widely expressed across diverse sensory neuron populations, including both myelinated sensory neurons and unmyelinated nociceptors.

### Tmem63b knockout mice exhibit reduced sensitivity to acute noxious heat

Given previous reports that recombinant TMEM63B can be activated by high-threshold mechanical stimuli [33,47] and its expression in large-diameter DRG neurons, we first assessed its potential role in mechanosensation. Behavioral analyses revealed that *Tmem63b*^*−/−*^ mice (KO) showed a nonsignificant trend toward reduced responsiveness to innocuous brushing stimuli compared to wild-type (WT) controls (Fig 2A). Quantitative testing with von Frey filaments demonstrated normal withdrawal responses to both innocuous and noxious mechanical stimuli in knockout animals (Fig 2B and 2C), suggesting TMEM63B is dispensable for basal mechanical pain perception.

**Fig 2:**
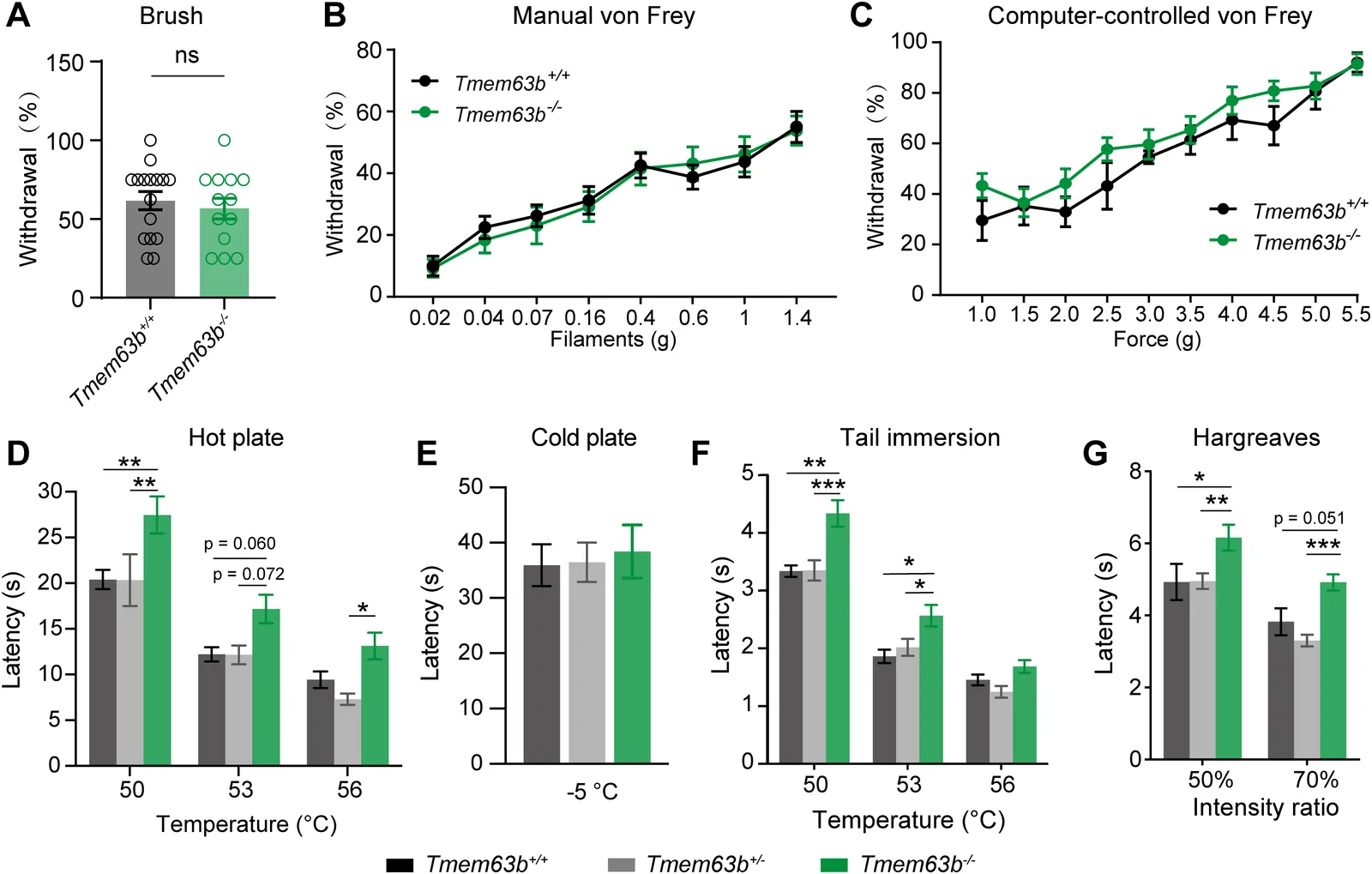
TMEM63B-deficient mice showed reduced sensitivity to acute noxious heat. **(A)** Brushing test of hind paw in WT mice (*n* = 16) and *Tmem63b* KO mice (*n* = 13). Unpaired *t* test. **(B)** Frequency of avoiding behaviors in response to different von Frey filaments in WT mice (*n* = 16), and *Tmem63b*^^*−*/*−*^^ mice (*n* = 13). **(C)** Paw withdrawal frequency to graded von Frey stimuli using an automated system in WT mice (*n* = 11), and *Tmem63b* KO mice (*n* = 13). **(D, E)** Response latency on the hot plate (D) or cold plate (E) in *Tmem63b*^*+/+*^ (*n* = 13), *Tmem63b*^^*−*/-*−*^^ (*n* = 17), *Tmem63b*^+/*−*^ (*n* = 11) mice. **(F, G)** Tail immersion (F) and Hargreaves (G) tests showed that *Tmem63b*^^*−*/*−*^^ mice exhibited impaired nociceptive responses. For tail immersion, *Tmem63b*^*+/+*^, *n* = 8 mice; *Tmem63b*^^*−*/*−*^^, *n* = 18 mice; *Tmem63b*^+/*−*^, *n* = 16 mice. For Hargreaves (50%), *Tmem63b*^*+/+*^, *n* = 12 mice; *Tmem63b*^^*−*/*−*^^, *n* = 21 mice; *Tmem63b*^+/*−*^, *n* = 18 mice. For Hargreaves (70%), *Tmem63b*^*+/+*^, *n* = 8 mice; *Tmem63b*^^*−*/*−*^^, *n* = 19 mice; *Tmem63b*^+/*−*^, *n* = 16 mice. For Fig 2B–2G, *p < 0.05; **p < 0.01; ***p < 0.001, two-way ANOVA analysis with Tukey's multiple comparisons test. Data are means ± SEM. The data underlying this Figure can be found in S1 Data.

We next examined whether TMEM63B is involved in thermosensation. In hot plate test, *Tmem63b*^*−/−*^ mice displayed impaired nociceptive responses at 50, 53, and 56 °C (Fig 2D). In contrast, they showed a normal response to a cold plate (−5 °C) compared to *WT* or *Tmem63b*^*+/−*^ mice (Fig 2E). In tail immersion test, *Tmem63b*^*−/−*^ mice exhibited significantly longer withdrawal latencies to hot water than *Tmem63b*^*+/−*^ and *Tmem63*^*+/+*^ mice (Fig 2F). Similarly, Hargreaves test showed that *Tmem63b*^*−/−*^ mice displayed impaired avoidance behavior (Fig 2G). These consistent phenotypic deficits specifically in heat sensation, but not in mechanical or cold responses, strongly implicate a role of TMEM63B in noxious thermosensation.

To verify that the impairment in noxious heat sensation was caused by the deficiency of TMEM63B in primary sensory neurons, we generated DRG-specific TMEM63B knockout mice (cKO, *Advillin*^*Cre/+*^; *Tmem63b*^*HA-fl/HA-fl*^, Fig 3A). Immunostaining confirmed efficient deletion, with positive neurons reduced from 52.1 ± 8.2% in *Tmem63b*^*HA-fl/HA-fl*^ mice to 4.6 ± 1.5% in the cKO mice (Fig 3B and 3C). Since there was no obvious difference in heat sensitivity between *Tmem63b*^*+/+*^ and *Tmem63b*^*+/−*^ mice (Fig 2D – 2G), we used the *Advillin*^*Cre/+*^*; Tmem63b*^*HA-fl/+*^ mice as a control, which retain one functional allele of *Tmem63b* in most DRG neurons. Hot plate tests showed that withdrawal latency responding to 50 and 53 °C was increased in cKO mice compared to control mice (Fig 3D). TMEM63B-cKO mice also exhibited slower avoidance responses in the tail immersion test and Hargreaves tests (Fig 3E and 3F). We further examined the thermal preference. The animals were allowed to move freely in a four-square platform set with different temperatures. Both cKO and control mice preferred to choose the innocuous warm temperature (33 and 38 °C) and avoid noxious heat (43 and 48 °C), but the tolerance to noxious heat was significantly increased in cKO mice (Fig 3G – 3I). Position reversal by setting temperatures (Scheme #1 and Scheme #2) demonstrated that spatial preference in mice was independent of the experimental environment. These data thus demonstrated that TMEM63B in DRG neurons is required for normal noxious heat sensation.

**Fig 3:**
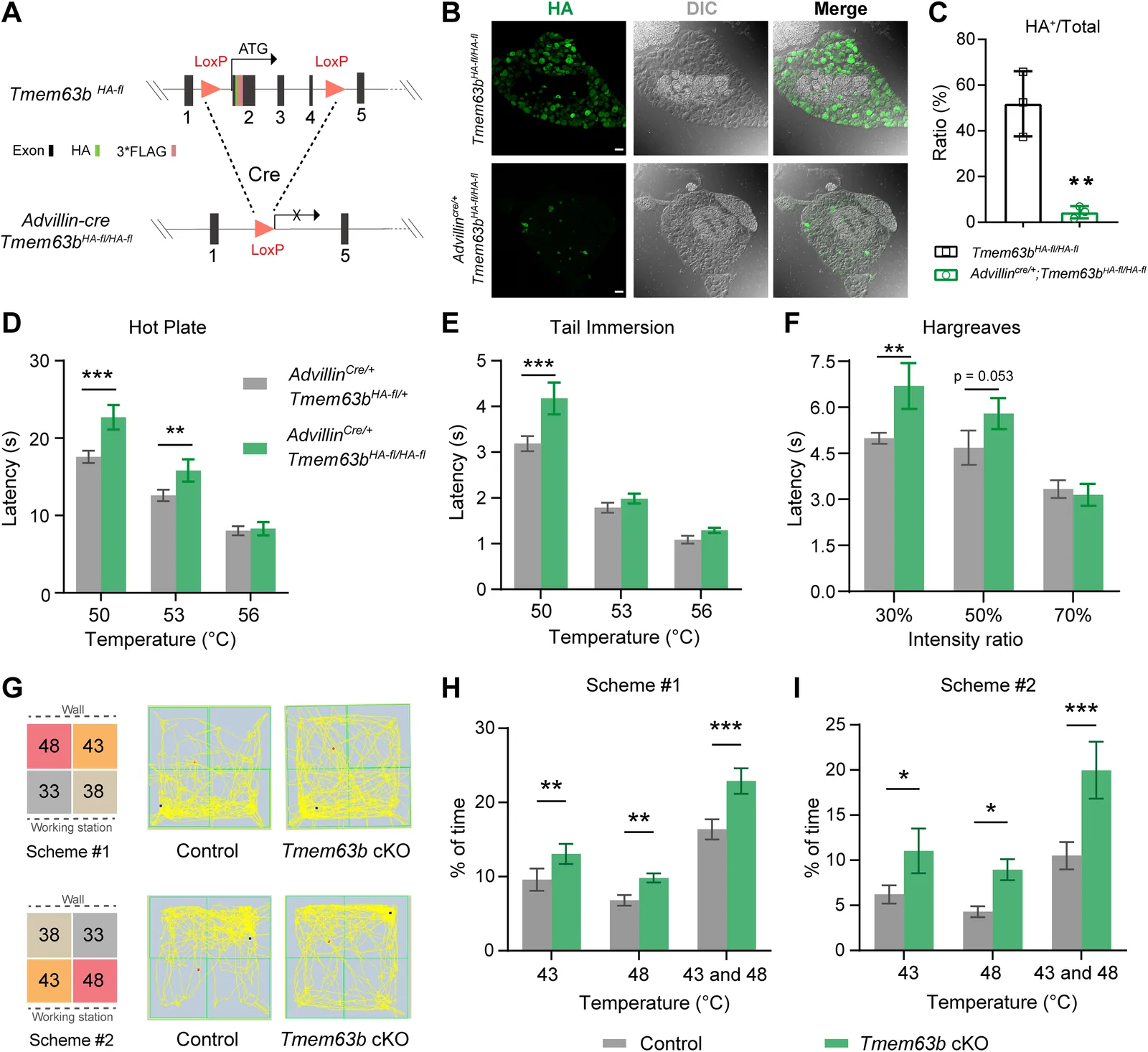
TMEM63B deletion in DRG neurons significantly impaired thermal nociception. **(A)** Strategy for generation of cKO mice using *Advillin-cre* and *Tmem63b*^*HA-fl/HA-fl*^ mice. **(B)** HA-Immunostaining images indicated that HA-TMEM63B signal (green) was absent in most of cKO DRG neurons. Scale bars, 50 μm. **(C)** Statistic ratio of HA-positive DRG neuron in cKO mice and control group. *n* = 3 mice, **p < 0.01, unpaired *t t*est. Data are means ± SEM. **(D–F)** Hot plate (D), tail immersion (E), and Hargreaves (F) tests showed that cKO mice displayed slower nociceptive responses. For hot plate, *n* = 11 mice in both cKO and control groups. For tail immersion and Hargreaves, control, *n* = 10 mice; cKO, *n* = 9 mice. **(G–I)** Four-temperature choice tests showed that cKO mice increased residence in the higher temperature (43 °C or 48 °C) area. Control and cKO, *n* = 13 mice. For Fig 3D–3I, *p < 0.05; **p < 0.01; ***p < 0.001; two-way ANOVA analysis with Tukey's multiple comparisons test. Data are means ± SEM. The data underlying this Figure can be found in S1 Data.

### TMEM63B deletion impairs heat-evoked Ca^2+^ signal in DRG neurons

To investigate the cellular mechanisms underlying TMEM63B-mediated heat sensation, we first examined whether TMEM63B deficiency affects DRG neuronal survival or subtype composition. Unlike its essential role in cochlear outer hair cell survival [30], TMEM63B knockout did not alter DRG neuronal structure or subtype distribution (S1 Fig), suggesting its function in sensory neurons is primarily physiological rather than developmental.

Given TMEM63B’s permeability to Ca^2+^ [30], we performed Ca^2+^ imaging on DRG neurons dissociated from *Tmem63b*^*+/−*^ and *Tmem63b*^*−/−*^ mice using Fluo-4 AM (Fig 4A and 4B). In responding to 43 °C, the proportion of heat-responding *Tmem63b*^*−/−*^ DRG neurons was reduced in neurons with virtually all different sizes (Fig 4C). Similarly, when exposed to 46 °C, fewer DRG neurons from *Tmem63b*^*-/-*^ mice responded to heat compared to those from *Tmem63b*^*+/−*^ mice across most size classes (Fig 4D). At 43 °C, ~27% of *Tmem63b*^*−/−*^ DRG neurons showed Ca^2+^ elevation, which was significantly lower than *Tmem63b*^*+/−*^ neurons (~42%, Fig 4E). Meanwhile, the amplitude of Ca^2+^ elevation was reduced in responding *Tmem63b*^*−/−*^ DRG neurons (Fig 4F). At 46 °C, both the proportion of heat-responsive neurons and the amplitude of Ca^2+^ elevation were significantly decreased in *Tmem63b*^*−/−*^ mice (Fig 4G and 4H). To examine the possibility that the reduced heat responsiveness was due to impaired neuronal viability or excitability, we quantified both the percentage of KCl-responsive neurons and the amplitude of KCl-evoked calcium transients. We found no differences between control and *Tmem63b*^*−/−*^ mice (S2 Fig), confirming that TMEM63B deficiency specifically affects thermal sensitivity rather than general neuronal health.

**Fig 4:**
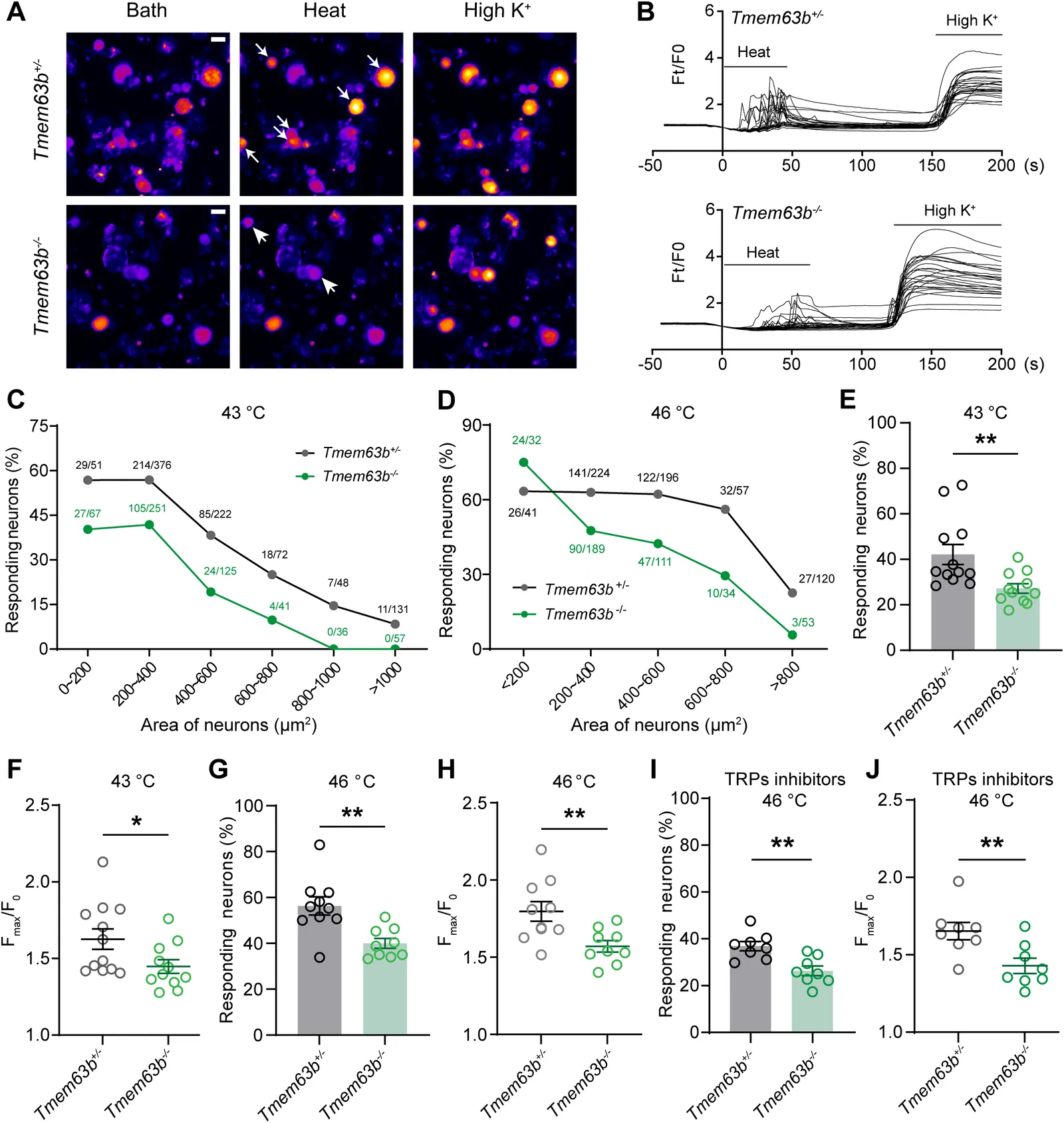
TMEM63B deletion impaired heat-evoked Ca^2+^ response of DRG neurons. **(A)** Calcium imaging of isolated DRG neurons responding to heat and K^+^ was monitored by Fluo-4 AM (left column). Scale bar, 30 μm. **(B)** Calcium intensity traces showed that *Tmem63b* deletion decreased response to heat (43 °C). **(C, D)** Response ratios of size-classified DRG neurons to 43 °C (C) and 46 °C (D). **(E)** Histogram represents total ratio of responding DRG neurons to 43 °C. *Tmem63b*^*+/−*^, *n* = 12 mice; *Tmem63b*^*−/−*^, *n* = 11 mice; **p < 0.01, unpaired *t t*est with Welch's correction. Data are shown as means ± SEM. **(F)** The fold changes in calcium fluorescence responding to 43 °C were calculated as F_max_/F_0_. The F_0_ represent the baseline fluorescent intensity at 0 s and F_max_ represent the maximum fluorescence intensity during heat stimulation. *p < 0.05, unpaired *t* tes*t* with Welch's correction. **(G)** Histogram represents total ratio of responding DRG neurons to 46 °C. *Tmem63b*^*+/−*^, *n* = 10 mice; *Tmem63b*^*−/−*^, *n* = 9 mice; **p < 0.01, unpaired *t* test. Da*t*a are shown as mean ± SEM. **(H)** The fold change in calcium fluorescence responding to 46 °C. **p < 0.01, unpaired *t* test. **(I)** Histogram represen*t*s total ratio of responding DRG neurons to 46 °C with inhibitors cocktail including 5 μM AMG9810, 100 μM HC030031, and 5 μM isosakuranetin. For *Tmem63b*^*+/−*^ and *Tmem63b*^*−/−*^, *n* = 8 mice. **p < 0.01, unpaired *t* test. Data are shown as mean ± SEM. **(J)** The fold change in calcium fluorescence responding to 46 °C wi*t*h TRPs inhibitors. **p < 0.01, unpaired *t* test. Data are shown as mean ± SEM. The data underlying this Figure can be found in S1 Data.

We next investigated whether TMEM63B modulates heat sensation through regulation of canonical TRP channels (TRPV1/M3/A1). We utilized cocktail inhibitors [26], including 5 μM AMG9810 (TRPV1), 100 μM HC030031 (TRPA1), and 5 μM isosakuranetin (TRPM3). To validate the effectiveness of the pharmacological blockade, we tested the responses of DRG neurons to high concentrations of specific agonists, 100 μM capsaicin (TRPV1), 100 μM pregnenolone sulfate (TRPM3), or 100 μM AITC (TRPA1), in the presence of the antagonist cocktail (S3 Fig). The cocktail profoundly inhibited agonist-evoked responses for all three TRP channels, almost abolishing their activation. In the presence of these antagonists, deletion of TMEM63B still significantly reduced heat-induced Ca^2+^ elevation in DRG neurons (Fig 4I and 4J). These results suggest that TMEM63B contributes to heat sensing independently of TRPV1, TRPM3, and TRPA1.

### TMEM63B deficiency in DRG neurons attenuates heat-evoked electrophysiological responses

We then studied the effects of TMEM63B on electrophysiological characteristics of DRG neurons. Because DRG neurons are highly heterogeneous in size and function, we chose to record neurons with diameters in the range of 18–25 μm to reduce the heterogeneity. We recorded heat-induced AP firing of DRG neurons with the temperature of the extracellular perfusion solution gradually increased from room temperature to 48 °C within 70–130 s (Fig 5A). Some control neurons began firing when temperature reached 37 °C, while APs were rarely observed in cKO DRG neurons before the temperature reached 43 °C (Fig 5B and 5C). The mean thermal activation threshold for heat-evoked APs was elevated by ~1.5 °C in TMEM63B-cKO neurons (Fig 5B). When heat stimuli were between 41 °C and 44 °C, the percentage of heat-excited DRG neurons was significantly reduced by TMEM63B deletion (Fig 5C). Analysis of the distribution of spike frequency throughout the heating temperature revealed that TMEM63B deletion mostly reduced DRG firing frequency between 41 and 43 °C (Fig 5D). We also observed that the resting membrane potential was unaltered by TMEM63B deletion (Fig 5E), suggesting that the basal activity of TMEM63B might be low in DRG neurons and does not contribute to the resting membrane potential.

**Fig 5:**
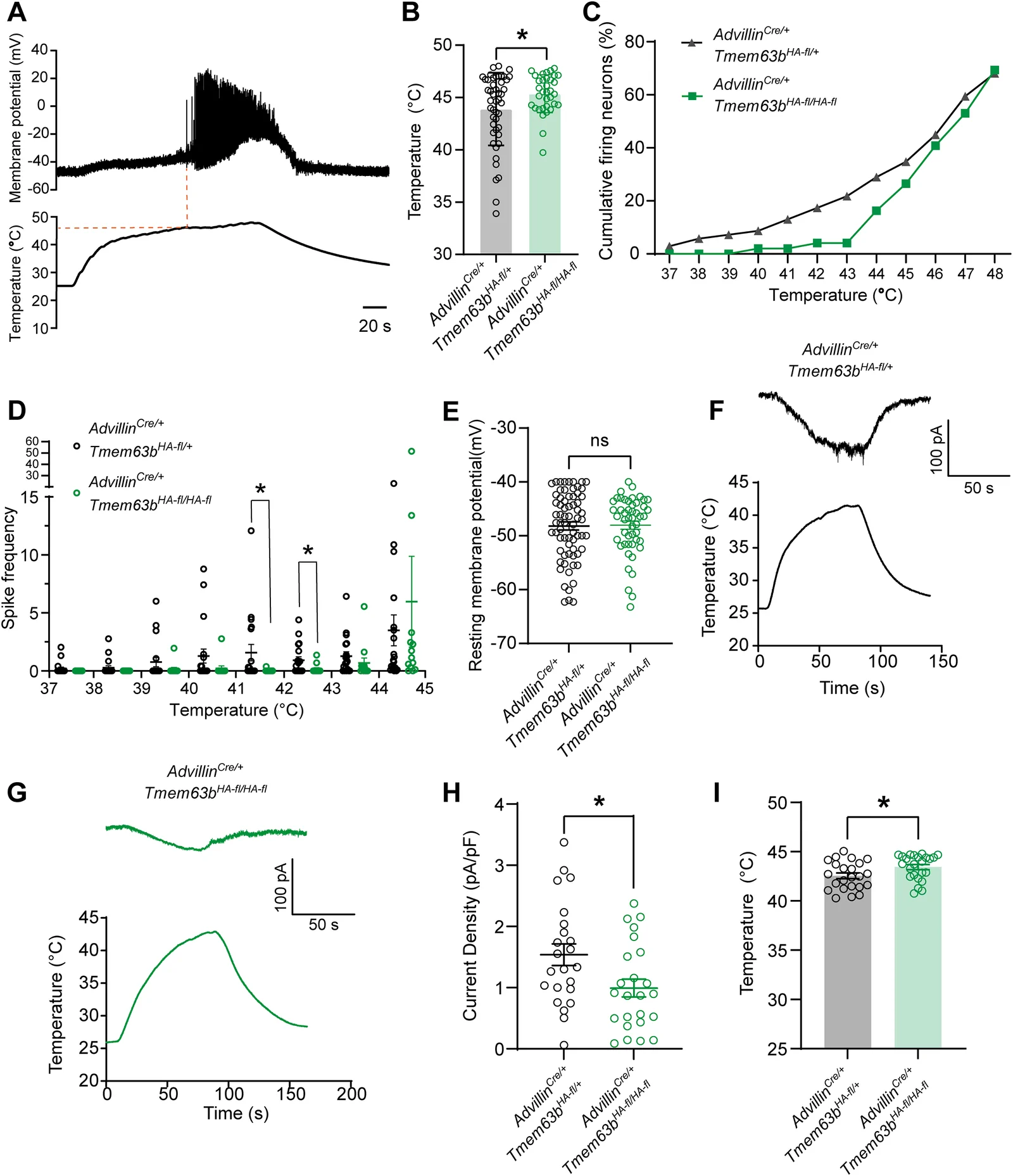
TMEM63B deletion in DRG neruons impaired heat-evoked electrophysiological responses. **(A)** AP firing in responding to heated extracellular solution. Temperature threshold was referred as the temperature excited the first spike. **(B)** Temperature threshold in control and cKO neurons. Control, *n* = 47 neurons; cKO, *n* = 34 neurons. *p < 0.05, unpaired *t* test with Welch's correction. Data are shown as mean ± SEM. **(C)** Accumulated percentages of firing neurons in responding to heat from 37 to 48 °C. Control, *n* = 69 neurons; cKO, *n* = 49 neurons. **(D)** Spike frequency from 37 to 45 °C in responding neurons. Data are shown as mean ± SEM; Control, *n* = 19 neurons; cKO, *n* = 13 neurons; Mann–Whitney test. *p < 0.05. **(E)** Resting membrane potentials of DRG neuron before heat stimulation. Control, *n* = 69 neurons; cKO, *n* = 49 neurons. Unpaired *t* tes*t*, ns represents no significance. **(F, G)** Whole-cell currents (V_holding_ at −60 mV) induced by heat in DRG neurons from control mice (F) and TMEM63B-cKO mice (G) in the presence of TRP antagonist cocktail. **(H)** Current density (pA/pF) analysis. **(I)** The temperature that heat-evoked currents reached plateau. For Fig 5H and 5I, *p < 0.05 with unpaired *t* test. Con*t*rol, *n* = 23 neurons; cKO, *n* = 24 neurons; Data are shown as mean ± SEM. The data underlying this Figure can be found in S1 Data.

To specifically evaluate the contribution of TMEM63B to thermosensitive currents, we performed voltage-clamp recordings (−60 mV holding potential) while pharmacologically blocking major heat-sensitive TRP channels using the antagonist cocktail described above [26]. Under this condition, DRG neurons still exhibited heat-evoked currents (Fig 5F and 5G). The current density was significantly reduced in DRG neurons from cKO mice compared with those from control mice (Fig 5H). The currents reached plateau at higher temperature in Tmem63b-cKO DRG neurons than in control neurons (Fig 5I).

Together, these results provide direct electrophysiological evidence that TMEM63B contributes to heat-sensitive currents independently of TRPV1, TRPM3, and TRPA1, consistent with its role as an essential molecular component for heat transduction in DRG neurons.

### Recombinant TMEM63B exhibited lipid-dependent thermosensitivity

We next examined whether recombinant TMEM63B is sensitive to thermal stimulation. TMEM63B was heterologously expressed together with the Ca^2+^ indicator GCaMP6f in mouse neuroblastoma N2a cells [30]. When perfused with extracellular solution heated to 46 °C, TMEM63B-expressing cells exhibited a very modest Ca^2+^ elevation compared to mock-transfected controls (Fig 6A, 6C, 6D).

**Fig 6:**
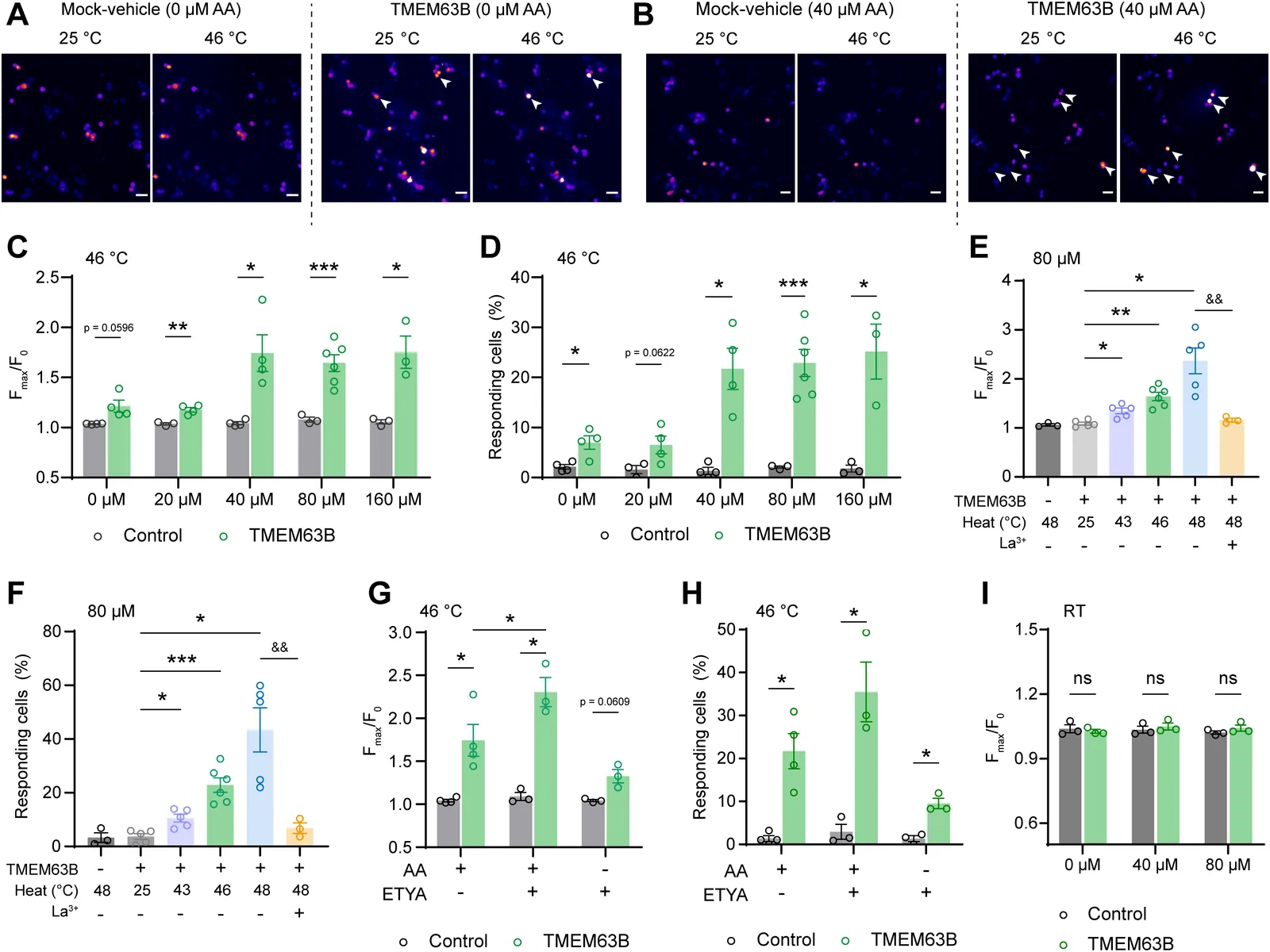
Recombinant TMEM63B mediated heat-evoke calcium raising especially in the presence of arachidonic acid. **(A)** Ca^2+^ imaging of N2a cells transfected with TMEM63B in response to 46 °C without AA. Scale bar, 30 μm. **(B)** Ca^2+^ imaging of N2a cells transfected with TMEM63B in the presence of 40 μM AA. Scale bar, 30 μm. **(C)** The fold changes of calcium fluorescent intensity (F_max_/F_0_) in response to heat stimulation in the presence of different concentrations of AA. **(D)** Ratio of responding cells in the presence of different concentrations of AA. *p < 0.05, **p < 0.01, ***p < 0.001, Tukey's multiple comparisons test. Data are shown as mean ± SEM. **(E)** The changes of calcium fluorescent intensity (F_max_/F_0_) stimulated by different temperatures in the presence of 80 μM AA. **(F)** Ratio of responding cells activated by different temperatures in the presence of 80 μM AA. *p < 0.05, **p < 0.01, ***p < 0.001, One-way ANOVA. ^&&^*p* < 0.01, Unpaired *t* test with Welch's correction. Data are shown as mean ± SEM. **(G)** The changes of calcium fluorescent intensity (F_max_/F_0_) stimulated by 46 °C in the presence of 40 μM AA or ETYA. **(H)** Ratio of responding cells evoked by 46 °C in the presence of 40 μM AA or ETYA. *p < 0.05, Tukey's multiple comparisons test. Data are shown as mean ± SEM. **(I)** The changes of calcium fluorescent intensity (F_max_/F_0_) in the presence of different concentrations of AA at room temperature. Tukey's multiple comparisons test. ns represents no significance. For all histograms, a dot represents one experiment. *n* = 3 ~ 6 experiments. The data underlying this Figure can be found in S1 Data.

Arachidonic acid (AA) is an endogenous unsaturated fatty acid known to activate some heat-sensitive channels such as TRPV4 [48,49] and TRAAK [50], or to lower the heat threshold of TRPV1 [51]. We therefore examined whether AA could enhance the thermosensitivity of TMEM63B. After pretreatment with 40 μM AA for 3~4 mins, noxious heat (46 °C) induced a robust Ca^2+^ rising in cells transfected with TMEM63B, but not mock-transfected cells (Fig 6B-6D). Concentrations of AA below 20 μM had little effects on heat-evoked calcium signals, whereas concentrations exceeding 40 μM consistently enhanced TMEM63B-mediated calcium responses to 46 °C stimulation (Fig 6C and 6D). At a constant AA concentration (80 μM), the Ca^2+^ fluorescence increased with heating temperature, which was abolished by 1 mM La^3+^ (Fig 6E and 6F), a broad-spectrum polycationic pore blocker that inhibits TMEM63B currents [30].

To verify the mechanism by which AA effects potentiate TMEM63B, we applied 5,8,11,13-eicosatetraynoic acid (ETYA), a nonmetabolizable analogue of AA that blocks AA metabolism [49,52,53]. ETYA (40 μM) did not antagonize the effects of AA on heat-induced Ca^2+^ signal; rather, it enhanced them. Indeed, ETYA alone also increased the proportion of TMEM63B-positive cells responding to 46 °C stimulation (Fig 6G and 6H). These findings suggest that AA potentiates TMEM63B through its own molecular properties rather than through downstream metabolites. Furthermore, at room temperature, AA induced no detectable Ca^2+^ signal in TMEM63B-transfected cells (Fig 6I), indicating that AA acts primarily as a sensitizer rather than a direct agonist.

In addition to its channel function, TMEM63B also possesses lipid scramblase activity that can alter cell membrane composition [34,35]. Since several TRP channels, such as TRPV1, TRPA1, and TRPM3, are known to be modulated by changes in the lipid environment [54–56], we investigated the possibility that the observed heat-evoked responses were contaminated by endogenous thermosensors in N2a cells. Treatment of GCaMP6f-transfected N2a cells with high concentrations of specific agonists for TRPV1 (capsaicin), TRPM3 (pregnenolone sulfate, PergS), and TRPA1 (AITC) evoked no detectable calcium influx (S4A–S4C Fig), confirming the absence of functional endogenous thermo-TRP channels in this cell line.

We previously demonstrated that TMEM63B channel is activated by hypoosmolality [30]. We therefore asked whether AA could also potentiate the osmosensitivity of TMEM63B. Reduction of the osmolarity of extracellular solution from 300 mOsm/L to 170 mOsm/L elicited Ca^2+^ elevation in TMEM63B- transfected N2a cells (S4D Fig). Both the amplitude of Ca^2+^ elevation and the proportion of responsive cells were significantly increased in the presence of 40 μM AA (S4E and S4F Fig). Therefore, AA potentiates both the thermosensitivity and osmosensitivity of TMEM63B.

We next performed whole-cell patch-clamp recordings to examine TMEM63B-mediated currents. Heat stimulation (48 °C) evoked currents that appeared slightly larger in TMEM63B-expressing cells compared to mock controls, although this difference did not reach statistical significance (*p* = 0.1417; Fig 7A – 7C). However, in the presence of 40 μM AA, heat-evoked currents were significantly potentiated in TMEM63B-expressing cells but not in mock-transfected cells (Fig 7D – 7H). These currents were blocked by ruthenium red (RR, 100 μM; S5G Fig). Application of 40 μM AA did not induce TMEM63B currents at room temperature (Fig 7I), further confirming its role as a potentiator rather than an agonist. To examine the possibility that TMEM63B acts by modulating other thermoreceptors, we co-expressed TMEM63B with TRPV1, TRPM3, or TRPA1. TMEM63B expression did not enhance agonist-induced currents (capsaicin, PregS, or AITC) for any of these channels (S5A–S5F Fig), indicating that TMEM63B's role in heat sensation is not mediated through potentiation of these known thermo-TRP channels.

**Fig 7:**
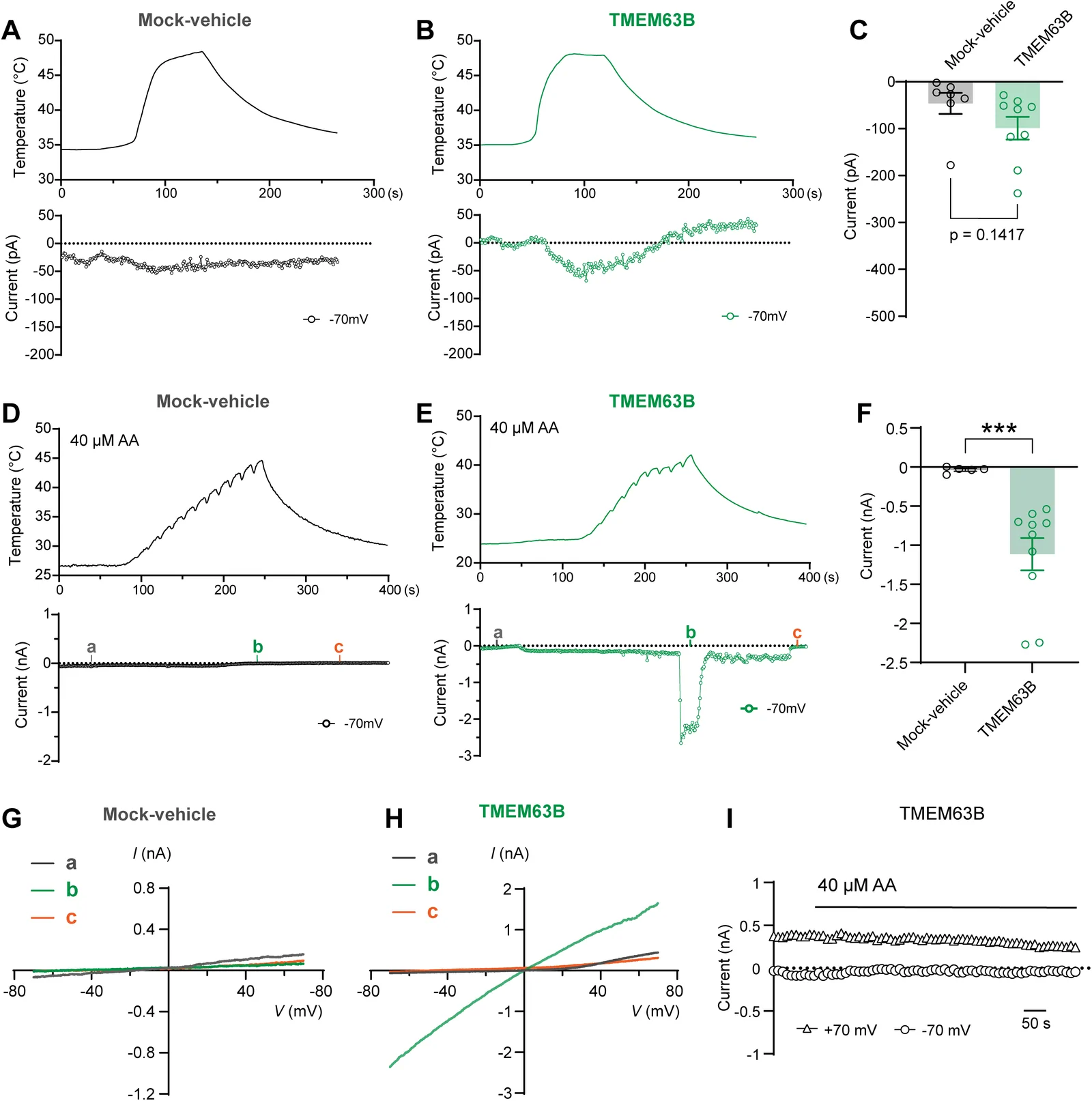
Recombinant TMEM63B mediated heat-induced currents in the presence of arachidonic acid. **(A, B)** Representative heat-induced currents at −70 mV in the mock cell (A) and TMEM63B-positive cell (B). **(C)** Current changes at peak temperature. Mock, *n* = 7 cells; TMEM63B, *n* = 9 cells. unpaired *t* test. Data are shown as mean ± SEM. **(D, E)** Representative heat-induced currents at −70 mV in the mock cell (D) and TMEM63B-positive cell (E) in the presence of 40 μM AA. **(F)** Current changes at peak temperature in the presence of 40 μM AA. Mock, *n* = 5 cells; TMEM63B, n = 10 cells. ***p < 0.001, unpaired *t t*est with Welch's correction. Data are shown as mean ± SEM. **(G)** Representative current (ramp protocol, −70 mV to 70 mV) related to time points from (D) in a mock cell. **(H)** Representative current related to time points from (E) in a TMEM63B-positive cell. **(I)** AA (40 μM) application did not induce current changes in TMEM63B-expressing cells at room temperature. The data underlying this Figure can be found in S1 Data.

Taken together, these data demonstrate that TMEM63B exhibits marginal heat-evoked response in heterologous systems, which is strongly potentiated by AA, revealing that its thermosensitivity is modulated by the membrane lipid environment.

## Discussion

This study identifies TMEM63B as an essential molecular component in DRG neurons that contributes to noxious thermosensation. Evidence supporting this notion includes: 1) TMEM63B is widely expressed across DRG neuronal subpopulations; 2) genetic ablation of TMEM63B impairs behavioral responses to noxious heat but not to mechanical pain; 3) TMEM63B deficiency reduces heat-evoked Ca^2+^ signals and current density, and elevates action potential thresholds in DRG neurons; and 4) recombinant TMEM63B exhibits a modest thermosensitivity that is potentiated by AA, revealing its dependence on the membrane lipid environment.

While TRPV1, TRPM3, and TRPA1 are well-established core components for noxious heat sensation [26], their triple knockout mice still exhibit heat avoidance in behavioral assays, indicating the existence of additional molecular components for heat sensation. Several candidates have emerged to account for this remaining heat sensitivity. TRPM2, traditionally characterized as a nonnoxious warm sensor [10,11], has also been shown to contribute to noxious heat perception. Vilar and colleagues demonstrated that a substantial proportion of neurons, after the pharmacological blockade of TRPV1, TRPM3, and TRPA1, remain heat-responsive, underscoring the role of TRPM2 in maintaining thermosensory redundancy [27]. This phenomenon closely mirrors our own calcium imaging observations (Fig 4I). For ANO1, while Takayama and colleagues initially proposed that it acts synergistically with TRPV1 as a secondary amplifier [29], subsequent work by Cho and colleagues revealed that ANO1 also possesses intrinsic heat sensitivity [28]. More recently, TMC6 has been shown to function as a GPCR-like receptor that senses noxious heat via the Gαq-PLC-IP3R-Ca^2+^ signaling pathway [23].

Compared to the intrinsic thermosensitivity of ANO1 and classical TRP channels such as TRPV1, TRPM3, and TRPM2, TMEM63B exhibits a distinct activation profile in heterologous systems. Its direct heat sensitivity is rather weak, which cannot be constantly observed in electrophysiological recordings and Ca^2+^ fluorescence imaging, but is dramatically potentiated by including AA at concentrations over 40 µM. In intact tissues, the basal level of free AA is typically reported to be in sub-micromolar ranges [57,58]; the plasma unesterified AA rapidly turnover in seconds [57,59]. Therefore, endogenous AA in DRG neurons unlikely reaches such high concentration at physiological conditions. Nevertheless, AA discovered here represents a lipid microenvironment facilitating TMEM63B thermosensitivity and osmosensitivity.

The gating mechanisms of TMEM63 family channels are not fully understood. Emerging evidence suggests that TMEM63B responds to membrane lipid remodeling. Structural studies reveal that OSCA/TMEM63 activation critically depends on lipid interactions within the protein crevice [60,61], where force-induced phospholipid reorganization may serve as a regulatory switch. This model is further supported by observations that OSCA/TMEM63 activation is triggered by changes in lipid composition [34,60,62], and that alterations in membrane lipid environments modulate mechanical sensitivity [63]. Additionally, TMEM63B may interact with binding partners such as SLC19A2, forming a heterodimer with Ca^2+^-dependent phospholipid scrambling activity [35]. Our finding that AA strongly facilitates TMEM63B thermosensitivity and osmosensitivity is consistent with this lipid-centric model. As a working hypothesis, we propose that thermal activation of TMEM63B channels may occur via heat-driven perturbations in lipid-protein interactions, ultimately promoting channel opening. One possible explanation for the weak thermosensitivity of recombinant TMEM63B is that heterologous expression systems may lack the optimal membrane lipid microenvironments or key binding partners required for robust thermal responses.

TMEM63B was initially characterized as a mechano- and osmo-sensitive cation channel [30,33]. However, we observed no difference in mechanical pain sensitivity between *WT* and *Tmem63b*^*−/−*^ mice, indicating that TMEM63B in DRG does not mediate noxious mechanosensation at basal conditions. Given the heterogeneity of DRG neurons and the broad expression of TMEM63B, it may function in subpopulation-specific mechanosensory processes, such as proprioception, mechanical itch, or texture perception [1,64–66]. Further studies are needed to explore these possibilities.

The TMEM63B-cKO mouse model provided an excellent tool to specifically investigate its physiological role in the DRG. However, the behavioral phenotypes of cKO mice were less pronounced than those of global knockout mice at 56 °C. We speculate that this discrepancy might arise from two possibilities. First, there is incomplete Cre-mediated deletion in the DRGs, as residual HA signals remained detectable (Fig 3B). This remaining TMEM63B population might partially compensate for the loss of function. Second, besides its peripheral distribution, TMEM63B is also expressed in the CNS [44,45,67]. We currently cannot rule out the possibility that CNS-expressed TMEM63B modulates central pain-processing circuitries in global knockout mice. Nevertheless, our cKO model demonstrates that DRG-expressed TMEM63B is critically involved in noxious thermosensation.

Several limitations of this study should be acknowledged. First, the evidence supporting a direct thermosensitivity of TMEM63B remains limited, as we only observed a very modest heat-responsiveness in heterologous expression systems. Second, and more importantly, our current experiments do not distinguish whether AA potentiates TMEM63B by modulating membrane properties or through a more direct binding mechanism. Polyunsaturated fatty acids are known to directly bind and allosterically modulate other ion channels such as TREK-1 [68]. Future structural studies using lipid nanodiscs and targeted mutagenesis will be essential to resolve this uncertainty. Furthermore, while our cKO model highlights that DRG-expressed TMEM63B plays an important role in noxious thermosensation, a cell-type specific rescue experiment would be needed to further illustrate the causal relationship between the DRG-specific deletion of TMEM63B and impaired heat sensation.

## Materials and methods

### Animal

All animal breeding and maintained at the animal facility of Model Animal Research Center of Nanjing University, Nanjing. All operations in animal experiments were conducted in accordance with the animal protocols (number: 2019-SY09), approved by the Institutional Animal Care and Use Committee at the animal facility of Model Animal Research Center of Nanjing University, Nanjing, China. *Tmem63b* knockout (*Tmem63b*^*−/-−*^) mice and *Tmem63b*^*HA-fl/HA-fl*^ mice were used in this study [30]. We crossed *Advillin-Cre* line [69] with *Tmem63b*^*HA-fl/HA-fl*^ mice to obtain cKO mouse line. Both male and female mice were used unbiasedly in all experiments.

### Immunofluorescent staining and TSA staining

Mice were anesthetized with CO_2_ and perfused with 0.1 M phosphate-buffered saline (PBS) and lumbar DRGs were dissected and collected carefully in cold PBS. Then they were fixed with 4% paraformaldehyde in PBS for 2–3 h at 4 °C. After washing with PBS, tissues were transferred into 30% sucrose solution in PBS at 4 °C overnight. Then they were immersed in optimal cutting temperature compound (O.C.T) embedding medium (SAKURA, Tissue-Tek, Japan) and subsequently frozen. Cryosections (~15 μm) were incubated in blocking solution contained with 10% FBS and 0.3% Triton X-100 in PBS at room temperature for 2 h. Subsequently, Tissues were incubated overnight with primary antibodies dissolved in blocking solution at 4 °C. After washing with PBST (PBS with 0.3% Triton X-100), these samples were incubated with specific secondary antibodies in 10% NHS and 0.3% Triton X-100 at room temperature for 2 h.

PFA-fixed, paraffin-embedded tissue sections (4 μm) were deparaffinized and rehydrated. For the first round of staining, heat-induced epitope retrieval (HIER) was performed by microwaving sections in AR6 buffer (Akoya Biosciences) according to the manufacturer’s instructions. After blocking endogenous peroxidase and nonspecific binding, sections were incubated with an anti-TRPV1 primary antibody (1:500) for 60 min at 37 °C, followed by incubation with an HRP-conjugated secondary antibody for 10 min. Fluorophore deposition was then catalyzed by applying Opal 650 tyramide working solution (Akoya Biosciences) for 10 min at room temperature in the dark. Slides were washed with TBST between all steps. To strip the primary and secondary antibody complexes from the first round and prepare for the second antigen, slides were subjected to a second HIER step in AR9 buffer (Akoya Biosciences) via microwave heating (100 °C for 1 min, followed by 95 °C for 10 min). This heating procedure effectively removed previously bound antibodies while preserving the covalently deposited Opal 650 fluorophore. For the second round of staining, sections were subsequently incubated with an anti-HA primary antibody (1:500), followed by the HRP-conjugated secondary antibody and Opal 570 tyramide working solution. Finally, nuclei were counterstained with DAPI, and slides were coverslipped using an anti-fade mounting solution.

Images were acquired by Zeiss LSM 800 or Leica SP5 confocal microscopy. Primary antibodies are rabbit anti-HA-Tag (1:500, #3724, Cell Signaling Technology), rabbit anti-Trpv1 (1:500, ACC-030, Alomone labs), goat anti-CGRP (1:500, AB36001, Abcam), and chicken anti-NFH (1:500, AB5539, Sigma), isolectin IB4 biotin conjugates (2 g/ml, I21414, Invitrogen). The secondary antibodies are donkey anti-rabbit, anti-mouse, anti-goat (488, 594, and 647nm) purchased from Thermo Fisher Scientific and goat anti-chicken 488, anti-guinea pig 633 IgGs from Abcam plc.

### Behavioral tests

All mice in behavioral experiment were 8–12 weeks old. Except for the tail immersion test, mice were allowed to acclimate for 1–2 h before the experiments.

Hot and cold plate tests. A transparent case was used to keep the animal on the surface of the plate (Ugo Basile product ID 35150). The temperature of the plate was set for cold test (5 °C) and hot test (50, 53, and 56 °C). The time of latency was defined as the period between the start point, when the animal is placed on the hot plate surface, and the end point, when the animal licks its paw or jumps off to avoid thermal pain. The latency was recorded with 3–5 trials per animal. The operation interval of each trial was 10 min. A cut-off latency of 60 s was set to avoid tissue damage.

Von Frey tests. Mice were placed on an elevated metal mesh floor. For the manual von Frey test, the filaments with varying diameters and stiffnesses were applied to the plantar surface of hind paw. The avoiding behaviors responding to each filament were recorded, each mouse underwent 5 trials with at least 8–10 min between consecutive tests. For automated von Frey test, we stimulated their hind paws with a series of forces (0.5–5.5 g) with a 0.5 mm von Frey filament using an automated dynamic plantar aesthesiometer (Ugo Basile product ID 37450), presented to the central plantar surface. 5 min interval was set between each trial. Withdrawing foot within 5 s was considered as positive outcomewith at least 8–10 min between consecutive tests.

Brush tests. Mice were placed upon an elevated wire mesh platform for at least 30 min allowed to habituate to the testing environment. A calligraphy brush was lightly swept across the plantar surface of the hind paw. A brisk paw withdrawal, flinching, or licking elicited by the brushing was recorded as a positive response. Each mouse was subjected to a total of 8 trials for each hind paw, with a inter-trial interval of 5 min to prevent sensitization.

Tail immersion test. Mice were restrained in a fixing apparatus. Their tail was exposed in water of targeted temperatures. The latency of tail-flick was recorded with 3–5 trials per animal. 10 min interval was set between each trial. The cut-off time was 15 s.

Hargreaves test. Mice were placed on an elevated transparent glass platform. We stimulated their hind paws with heat provided by small incandescent light bulbs (Techman, PL200). The light intensity was adjusted to a level that *WT* mice withdraw their hind paw within 9-12s. The latency to hind paw withdrawal was recorded with 3 trials per animal. The operation interval of each trial was 10 min. A cut-off latency of 15 s will be set to avoid tissue damage.

Four-temperature choice test. The mice for test were free to move in the field consist of four independent hot plates (15 * 15 cm) with different temperature (33,38, 43, and 48 °C). The route and time of mice were recorded for 10 min.

### Cell culture

Neuro2a (N2a) cells were grown in Dulbecco’s Modified Eagle Medium (DMEM) containing 10% fetal bovine serum (Gibco, USA) at 37 °C with 5% CO2.

For culture of DRG neurons, lumbar DRGs were dissected and collected carefully in cold DF medium (a mixture of DMEM/F-12 basal medium, 10% fetal bovine serum, 100 U/ml of penicillin/streptomycin). Then they were digested by enzyme solution (1.5 mg/ml Collagenase II and 2 mg/ml Dispase II, sigma, in DF medium) at 37 °C for 30 min. DRGs were washed with HBSS (without Ca^2+^ and Mg^2+^), followed by mechanical dissociation in DF medium. After filtration and centrifugation, cells were plated on coverslips coated with poly-D-lysine (0.1 mg/ml, sigma) and laminin (50 μg/ml, sigma), and cultured in DF+ medium (DF medium added 2% B27, Invitrogen) in an 37 °C incubator with 5% CO_2_ for 3–4 hours. For AP recording experiments, we would oxygenate the DF medium in advance and added NGF (1.5 mg/ml, Invitrogen) to the DF+ medium. The neurons were used after 4–6 hours for calcium imaging, and for AP recording they were used after 12–20 hours.

### Calcium imaging

For N2a cells, the cytoplasmic level of calcium was monitored by using GCaMP6f, an indicator of free calcium. The TMEM63B-P2A-mCherry and GCaMP6f vectors was transfected simultaneously for 24–30 h into the N2a cells grown on the coverslips coated by poly-D-lysine (0.1 mg/ml, sigma). Cells that transfected with mCherry and GCaMP6f vectors were used as a control. For cultured DRG neurons, the cytoplasmic level of calcium was monitored by using the indicator Fluo-4 AM. Cells were stained in medium without serum. DRGs would be incubation in DF+ medium without serum contained Fluo-4 AM (5 μM, Invitrogen) for 30 min. Then they were balanced in DF+ medium without dye for 10–20 min. Cells were perfused with extracellular solutions heated by a Warner TC-324C heater. The temperature was increased within 60s. Extracellular solutions contained (mM): 140 NaCl, 5 KCl, 2 CaCl_2_, 1 MgCl_2_, 10 HEPES, pH 7.4 adjusted with NaOH, osmolarity adjusted by glucose to 300 mOsm/L. High K^+^ solution contained (mM): 100 NaCl, 50 KCl, 2 CaCl_2_, 1 MgCl_2_, 10 HEPES, pH 7.4 adjusted with NaOH. To eliminate artifactual calcium fluctuations, heat-evoked intracellular Ca^2+^ signals exhibiting 50% amplitude elevation relative to baseline were classified as positive responses, consistent with published methodologies [30].

### Electrophysiological recording

The TMEM63B-P2A-mCherry plasmid was transfected into the cell line. After transfection, the cells were digested into single cells and cultured on the cover glass covered by poly-D for 10 hours. The whole-cell current was recorded using 3–8 MΩ glass pipettes. The pipettes solution contains (mM): 140 K-gluconate, 1 Mg-gluconate, 5 EGTA, 10 HEPES. The pH was adjusted to 7.4 by KOH. Extracellular solution contains (mM): 135 Na-gluconate, 2 Ca-gluconate, 1 Mg-gluconate, 10 HEPES, 5 glucoses, pH was adjusted to 7.4 by NaOH. Osmotic pressure was adjusted to 300 mOsm/L with mannitol. Cells were maintained at −70 mV when the whole-cell patch clamp recording pattern was formed. Discarded the cells that membrane resistance was less than 400 MΩ. Recording program was set to give a ramp voltage from −70 mV to +70 mV within 70 ms. A Warner TC-324C heater was used to heating extracellular solutions. The temperature was increased within 60 s. Stimuli solutions were administered through a gravity irrigation system.

The AP of the DRG neurons were recorded under the current clamp. At first in the voltage clamp mode, the cell forms a whole-cell recording mode at −60 mV holding potential. Discarded the cells that membrane resistance was less than 300 MΩ. Then, switch the mode to current clamp, where cells were remained at current-free. Heat stimulation was recorded at temperatures ranging from room temperature (22–25 °C) to noxious temperature (45–48 °C) within 70 to 130 s. Discarded cells with a resting membrane potential higher than −40 mV. The pipette solution contains (mM): 126 K-gluconate, 10 NaCl, 1 MgCl_2_, 10 EGTA, 10 HEPES, 2 Na_2_-ATP, 0.5 Na_3_-GTP, adjusted to pH 7.4 with KOH and osmolarity to 295–300 mOsm with mannitol. For heat-evoke currents recording, the DRG neurons were recorded under the voltage clamp at −60 mV holding potential. The pipette solution contains (mM): 140 CsCl, 2 MgCl_2_, 5 EGTA, 10 HEPES, 2 Na_2_-ATP, 0.5 Na_3_-GTP, adjusted to pH 7.4 with CsOH and osmolarity to 295–300 mOsm with mannitol. All extracellular solution contained (mM): 138 NaCl, 5 KCl, 2 CaCl_2_, 1 MgCl_2_, 10 HEPES, 10 glucoses, adjusted pH to 7.4 with NaOH and osmolarity to 295–300 mOsm with mannitol.

### Statistical analysis

In this paper, ImageJ (Fiji) software was used to analyze the fluorescence gray value and DRG cross-sectional area. GraphPad Prism 10.3 was used for difference analysis. For electrophysiological experiments, data were collected and analyzed by pCLAMP10 and Clampfit 10.4 software. Group differences in calcium and currents analysis were assessed using unpaired *t*-tests or ANOVA tests. When unequal variances were detected by F-tests (*p* < 0.05), Welch’s correction was applied. For behaviors test, the *t*-*t*ests, one-way ANOVA or two-way ANOVA analysis of variance was employed for multigroup analyses. All experiments were repeated for three or more times, and the detailed information has been shown in the figure legend.

## Supporting information

S1 Fig TMEM63B deletion did not alter composition of DRG neurons. **(A)** Immunofluorescence staining of classic DRG markers (Green). Red signals show neurons labeled by neuN. Scale bar, 50 μm. **(B)** Percentage of marker-positive neurons in KO and WT mice. KO mice showed normal distribution of cell subsets labeled by classic DRG markers. n = 700 ~ 1,200 neurons from more than 3 mice. The data underlying this Figure can be found in S1 Data.
(TIF)

S2 Fig *Tmem63b* deletion did not alter the calcium response to KCl in DRG neurons. (A) The fold change in calcium fluorescence responding to KCl. (B) Histogram represents the total ratio of responding DRG neurons to KCl. Unpaired *t* test, *n* = 10 mice, no significance, Unpaired *t* test. Data are shown as mean ± SEM. The data underlying this Figure can be found in S1 Data.
(TIF)

S3 Fig Testing of the effective blockade of TRP channels in DRG neurons by calcium imaging. (**A–C)** Representative calcium imaging traces showing the responses of DRG neurons to 100 μM AITC (A), 100 μM capsaicin (B), and 100 μM pregnenolone sulfate (C), in the absence or presence of the TRP channel inhibitor cocktail. **(D–F)** Quantification of the percentage of responding cells, demonstrating the significant inhibitory effects of the cocktail against AITC (D), capsaicin (E), and PregS (F) induced calcium responses. Data are presented as mean ± SEM. ***p < 0.001, unpaired *t* test. *n* = 3–4 mice. The data underlying this Figure can be found in S1 Data.
(TIF)

S4 Fig Calcium responses of N2a cells to TRP channel agonists and AA-induced potentiation of TMEM63B osmosensitivity. **(A–C)** Representative images and corresponding calcium imaging traces of GCaMP6-expressing N2a cells in response to 1 mM AITC (A), 1 mM capsaicin (B), and 1 mM PregS (C). Scale bar: 100 μm. (D) Schematic of enhanced calcium fluorescence in N2a cells treated with 40 μM arachidonic acid (AA), demonstrating potentiated osmosensitivity of TMEM63B. Scale bar: 30 μm. (E) Fold change in Ca^2+^ fluorescence intensity (F_max_/F₀) under 170 mOsm/L hypotonic stress with 40 μM AA. ***p < 0.001, One-way ANOVA. (F) Proportion of responsive cells. *p < 0.05, ***p < 0.001, One-way ANOVA. The data underlying this Figure can be found in S1 Data.
(TIF)

S5 Fig TMEM63B does not affect TRP channel activity. **(A)** Representative traces showing the absence of agonist-induced inward currents in mock-transfected cells. **(B)** Representative traces showing inward currents successfully evoked by their respective agonists in cells individually expressing TRPA1, TRPV1, or TRPM3. **(C)** Representative traces of agonist-evoked inward currents in cells co-expressing individual TRP channels with TMEM63B. **(D–F)** Quantification of current amplitudes reveals that the co-expression of TMEM63B does not alter the inward currents mediated by TRPA1 (D), TRPV1 (E), and TRPM3 (F). ns represents no significance, Unpaired *t* test. Data are shown as mean ± SEM. (G) In the presence of 40 μM AA, heat-evoked current in a TMEM63B-positive cell was blocked by 100 μM RR. The data underlying this Figure can be found in S1 Data.
(TIF)

S1 Data Experimental raw data used for all figures in this study. This table provides the complete dataset and the exact experimental conditions that underlying the results presented in Figs 1–7 and S1–S5.
(XLSX)

## Abbreviations

AA: Arachidonic acid
ASICs: acid-sensing ion channels
cKO: conditional knockout
CNS: central nervous system
DMEM: Dulbecco’s Modified Eagle Medium
DRG: dorsal root ganglia
HIER: heat-induced epitope retrieval
PBS: phosphate-buffered saline
RTKs: receptor tyrosine kinases
TRP: transient receptor potential
WT: wild-type.
